# Assessment of the correlation between KAP scores regarding sugar-sweetened beverage consumption and hyperuricemia amongst Chinese young adults

**DOI:** 10.1186/s12889-024-18513-x

**Published:** 2024-04-17

**Authors:** Yun Zhang, Hong Di, Juan Wu, Xiaoxue Wang, Xinxin Han, Bingqing Zhang, Xuejun Zeng

**Affiliations:** Department of Family Medicine, Division of General Internal Medicine, Department of Medicine, Peking Union Medical College Hospital, State Key Laboratory of Complex Severe and Rare Diseases (Peking Union Medical College Hospital), Chinese Academy of Medical Sciences, No.1 Shuaifuyuan, Dongcheng District, 100730 Beijing, China

**Keywords:** Sweetened drink, Soft drink, Knowledge, attitude, practice, Hyperuricemia, China

## Abstract

**Background:**

The prevalence of hyperuricemia in China has been consistently increasing, particularly among the younger generation. The excessive consumption of sugar-sweetened beverages is associated with hyperuricemia. This study examined the knowledge, attitudes, and practices (KAP) of Chinese young adults regarding sugar-sweetened beverage consumption and the correlation with hyperuricemia.

**Methods:**

This cross-sectional investigation was conducted from June 28th, 2023, to July 21st, 2023, and enrolled Chinese young adults. Demographics and KAP were evaluated using a questionnaire (Cronbach’s α = 0.787). Factors influencing KAP scores were analyzed using multivariable analyses.

**Results:**

A total of 1288 valid questionnaires were analyzed. The median knowledge, attitude, and practice scores were 16 (12,19)/22, 22 (20,24)/30, and 27.5 (23,31.75)/40. The multivariable analysis showed that bachelor’s/associate education (OR = 1.912, 95%CI: 1.128–3.239), white collar/employee (OR = 0.147, 95%CI: 0.105–0.206), educator (OR = 0.300, 95%CI: 0.174–0.518), healthcare worker (OR = 0.277, 95%CI: 0.188–0.407), not suffering from hyperuricemia (OR = 0.386, 95%CI: 0.253–0.590), and not having gout (OR = 0.456, 95%CI: 0.282–0.736) were independently associated with knowledge. Age 26–30 (OR = 1.470, 95%CI: 1.052–2.052), age 31–35 (OR = 1.489, 95%CI: 1.097–2.022), age 36–40 (OR = 0.328, 95%CI: 1.010–1.746), age 41–44 (OR = 1.548, 95%CI: 1.091–2.198), and not having hyperuricemia (OR = 0.512, 95%CI: 0.345–0.760) were independently associated with attitude. White collar/employee (OR = 0.386, 95%CI: 0.285–0.521), educator (OR = 0.534, 95%CI: 0.317–0.899), healthcare worker (OR = 0.341, 95%CI: 0.236–0.493), having siblings (OR = 0.725, 95%CI: 0.573–0.917), and not suffering from hyperuricemia (OR = 0.442, 95%CI: 0.296–0.659), were independently associated with practice.

**Conclusion:**

Chinese young adults display moderate KAP toward sugar-sweetened beverages. Notably, an association was observed between hyperuricemia and each KAP dimension.

**Supplementary Information:**

The online version contains supplementary material available at 10.1186/s12889-024-18513-x.

## Background

Hyperuricemia refers to an elevated concentration of uric acid in the blood (i.e., blood uric acid ≥ 420 µmol/L), potentially leading to gout and nephrolithiasis [1, 2]. Hyperuricemia results from the interplay of increased uric acid production (e.g., purine-rich diet, enzyme deficiencies, and cell breakdown/turnover) and excretion (e.g., chronic kidney disease, acidosis, and medications) [2, 3]. In recent years, hyperuricemia has emerged as a significant global public health concern since it is estimated that 21% of the general population and 25% of hospitalized patients have asymptomatic hyperuricemia [4, 5]. The prevalence of hyperuricemia has been steadily increasing in China, particularly among the younger generation [6]. Fructose is increasingly recognized as an underlying cause of hyperuricemia [7, 8]. Indeed, fructose metabolism generates uric acid salts as byproducts, contributing to hyperuricemia [7–9]. Moreover, such metabolism perturbations can give rise to other metabolic disorders, including dyslipidemia and hyperglycemia, which are also public health issues [10, 11]. Sugar-sweetened beverages, replete with substantial fructose content, have thus emerged as pivotal factors linked to hyperuricemia [12, 13].

Drinking or not sweetened beverages is part of everyday life habits, and proper knowledge of the potential harms is necessary to adopt the proper habits. A knowledge, attitude, and practice (KAP) survey is a structured survey methodology that was traditionally extensively used in sociology and psychology, and nowadays, it is more and more used in the medical domain. KAP surveys allow the identification of knowledge gaps, misconceptions, and misunderstandings that constitute barriers to correctly implementing a specific subject in a specific population [14, 15]. KAP can provide elementary information for designing and refining health education and disease management strategies. While KAP studies have already explored hyperuricemia [16] and one study in Mexico examined sugar-sweetened beverage consumption and the risk of hyperuricemia [12], no data is available regarding the relationship between the KAP pertaining to sugar-sweetened beverage consumption and the correlation with hyperuricemia in Chinese young adults.

Therefore, this study aimed to examine the KAP toward sugar-sweetened beverages and its correlation with hyperuricemia among Chinese young adults. The results could lay the groundwork for more tailored health education initiatives.

## Methods

### Study design and participants

This cross-sectional investigation was conducted from June 28th, 2023, to July 21st, 2023, and enrolled Chinese young adults. The study was approved by the Ethics Committee of Peking Union Medical College Hospital affiliated to the Chinese Academy of Medical Sciences (approval #I-23ZM0019). Written informed consent was obtained from all participants.

The inclusion criteria were (1) voluntary participation and (2) individuals aged 14 to 44 years. The exclusion criteria were (1) age mismatch and (2) inability to complete the electronic questionnaire. The study was conducted by convenient sampling. The participants were recruited from the gout clinic of Peking Union Medical College Hospital, the public WeChat account of the gout clinic, and the medical examination center of hospitals in Shanghai, Jiangsu, Guangdong, Sichuan, Xinjiang, Guizhou, Hebei, and other cities. The questionnaire was developed using the Wenjuanxing (Questionnaire Star https://www.wjx.cn/app/survey.aspx) app. The participants accessed the questionnaire and responded to it via a QR code.

### Questionnaire development

The questionnaire design adhered to the pertinent guidelines and literature [12, 17–21]. After the preliminary questionnaire was finalized, two small-scale pre-experiments were conducted. The first pre-experiment was in 51 participants and revealed a Cronbach’s α of 0.680. According to the results of the first pre-experiment, the questionnaire was fine-tuned regarding the wording of attitude and practice options. The second pre-experiment was performed in 40 participants and revealed a Cronbach’s α of 0.787.

The questionnaire encompassed four sections: demographic data, knowledge dimension, attitude dimension, and practice dimension. For a detailed questionnaire, please refer to supplementary file. The knowledge dimension comprises 14 questions within two categories: 12 single-choice questions scored 1 for correct answers and 0 for incorrect or unclear responses and two multiple-choice questions with each correct option awarded 1 point. The knowledge component scores ranged from 0 to 22. The attitude dimension had seven questions, of which six were scored using a 5-point Likert scale, ranging from “extremely positive” (5 points) to “extremely negative” (1 point), yielding scores between 6 and 30. The practice dimension comprised 11 questions, with nine being scoring items and scores spanning 7 to 40. Likert scales provide a convenient way to measure unobservable constructs, and published tutorials detailing the process of their development have been highly influential [22, 23].

### Statistical analysis

The distribution of the scores within each dimension was examined for normal distribution. Non-normally distributed data were presented using median, 25th percentile, and 75th percentile and analyzed using the Wilcoxon-Mann-Whitney test (comparison of two groups) or the Kruskal-Wallis analysis of variance (comparison of more than two groups). The correlation between scores across dimensions was assessed using the Spearman correlation coefficient. The results were categorized based on the median scores within each dimension. The variables with *P* < 0.10 in the univariable analyses were included in the multivariable regression analyses. Two-sided *P*-values < 0.05 were considered statistically significant.

## Results

### Characteristics of the study population

A total of 1288 valid questionnaires were collected and analyzed. Most participants were between 19 and 40 years of age (82.3%). The gender distribution was predominantly female (56.6%). The educational levels of participants were predominantly with a bachelor’s degree or above (93.4%). Among the participants, 15.8% were students, 37.4% were white-collar/company employees, 5.8% were educators, and 27.1% were healthcare workers. Most participants had siblings (53.4%). Body weight distribution was mainly categorized as normal (46.7%) and overweight (29.5%). The study included 253 participants with hyperuricemia, accounting for 19.2%, and 183 participants with gout, accounting for 14.2% (Table 1).

**Table 1:** Baseline participant information and scores in KAP dimensions

	n (%)	Knowledge		Attitude		Practice	
		Median (P25, P75)	*P*	Median (P25, P75)	*P*	Median (P25, P75)	*P*
Total	1288	16 (12, 19)		22 (20, 24)		27.5 (23, 31.75)	
Gender			0.141		0.403		0.225
Male	559 (43.4)	16 (11, 19)		22 (20, 24)		27 (23, 31.5)	
Female	729 (56.6)	16 (12, 20)		22 (20, 24)		28 (23, 32)	
Age			0.145		< 0.001		< 0.001
< 15 years	2 (0.2)	9.5 (9, 10)		19 (18, 20)		20.5 (14, 27)	
15–18 years	11 (0.9)	14 (10, 17)		22 (21, 24)		28 (23, 32)	
19–25 years	270 (21.0)	16 (12, 19)		21 (19, 23)		25.25 (21, 30)	
26–30 years	296 (23.0)	16 (13, 19)		22 (20, 23.5)		28 (23.5, 32)	
31–35 years	249 (19.3)	16 (12, 19)		22 (20, 24)		28 (24, 32)	
36–40 years	296 (23.0)	16 (12, 20)		22 (20, 24)		27 (23, 31.75)	
41–44 years	164 (12.6)	15 (11.5, 20)		23 (21, 25)		29 (24.5, 33.5)	
Education			< 0.001		0.790		0.025
Primary school	1 (0.1)	9 (9, 9)		20 (20, 20)		27 (27, 27)	
Junior high school	33 (2.6)	10 (8, 13)		22 (20, 25)		24.5 (21, 28)	
High school	50 (3.9)	14 (10, 17)		22 (20, 24)		25.75 (21, 31)	
Bachelor’s/associate’s	731 (56.8)	16 (12, 19)		22 (20, 24)		27.5 (23, 31.5)	
Postgraduate and above	473 (36.6)	16 (13, 20)		22 (20, 24)		28 (23, 32)	
Occupation			< 0.001		< 0.001		< 0.001
Student	203 (15.8)	15 (11, 18)		21 (20, 23)		25 (21, 30)	
White-collar/employee	476 (37.0)	14 (11, 18)		22 (20, 24)		27 (23, 30.75)	
Educator	75 (5.8)	16 (11, 19)		22 (20, 24)		28.5 (23, 33)	
Healthcare worker	349 (27.1)	19 (16, 21)		22 (20, 24)		30 (25.5, 33)	
Other	185 (14.3)	15 (11, 18)		22 (21, 24)		27 (22.5, 31.5)	
Are you the only child?			0.012		0.964		0.004
Yes	600 (46.6)	17 (13, 20)		22 (20, 24)		28 (24, 32)	
No	688 (53.4)	16 (11, 19)		22 (20, 24)		27 (23, 31.5)	
Weight status			0.002		0.009		0.003
Underweight	145 (11.3)	15 (10, 18)		21 (20, 23)		25 (21.5, 30.5)	
Normal weight	601 (46.7)	16 (12, 19)		22 (20, 24)		28 (24, 32)	
Overweight	380 (29.5)	16 (12, 19)		22 (20, 24)		27.25 (23, 32)	
Obese	162 (12.5)	17 (13, 20)		22 (20, 25)		27.5 (24, 32)	
Do you suffer from hyperuricemia?			< 0.001		< 0.001		< 0.001
Yes	253 (19.6)	19 (15, 20)		23 (21, 25)		30.5 (26.5, 33.5)	
No	1035 (80.4)	15 (11, 19)		22 (20, 24)		27 (22.5, 31)	
Do you suffer from gout?			< 0.001		< 0.001		< 0.001
Yes	183 (14.2)	18 (15, 20)		23 (21, 25)		29.5 (26, 34)	
No	1105 (85.8)	16 (12, 19)		22 (20, 24)		27 (23, 31)	

### Knowledge, attitudes, and practices scores

The average knowledge score was 16 (12, 19) out of a total of 22 points (72.7%) (Table 1). Among the participants, 72.7% had heard of hyperuricemia, 47.0% were aware of the triggering factors of hyperuricemia, 51.5% were aware of the risk factors of hyperuricemia, 59.2% were aware that the consumption of sweetened beverages was associated with hyperuricemia, 46.8% were aware that fructose was responsible for hyperuricemia, 47.1% knew that purine levels in sweetened beverages were not related to hyperuricemia, 71.8% knew that hyperuricemia was related to gout, 56.0% were aware that hyperuricemia increases the risk of diabetes, 65.2% knew that hyperuricemia was associated with kidney diseases, and 68.7% were familiar with the sugar content of beverages (Tables 2 and 3).

**Table 2:** Responses to items in the participant knowledge dimension

	a. Yes (1 point)	b. No (0 points)	c. Uncertain (0 points)
Have you heard of hyperuricemia?	936 (72.7)	262 (20.3)	90 (7.0)
	a. Yes (1 point)	b. No (0 points)	c. Uncertain (0 points)
Are you aware of the triggering factors of hyperuricemia?	605 (47.0)	483 (37.5)	200 (15.5)
	a. Yes (1 point)	b. No (0 points)	c. Uncertain (0 points)
Do you comprehend the risks associated with hyperuricemia?	662 (51.4)	467 (36.3)	159 (12.3)
	a. Yes (1 point)	b. No (0 points)	c. Uncertain (0 points)
Is the intake of sugary beverages related to hyperuricemia?	762 (59.2)	41 (3.2)	485 (37.6)
	a. Yes (1 point)	b. No (0 points)	c. Uncertain (0 points)
Is fructose in sugary beverages a significant factor in causing hyperuricemia?	603 (46.8)	56 (4.4)	629 (48.8)
	a. Yes (0 points)	b. No (1 point)	c. Uncertain (0 points)
Are low purine levels in sugary beverages unlikely to cause hyperuricemia?	110 (8.5)	607 (47.1)	571 (44.4)
	a. Yes (1 point)	b. No (0 points)	c. Uncertain (0 points)
Is hyperuricemia closely related to the onset of gout?	925 (71.8)	16 (1.3)	347 (26.9)
	a. Yes (1 point)	b. No (0 points)	c. Uncertain (0 points)
Does hyperuricemia increase the risk of developing diabetes?	722 (56.0)	32 (2.5)	534 (41.5)
	a. Yes (1 point)	b. No (0 points)	c. Uncertain (0 points)
Can severe hyperuricemia lead to acute kidney failure?	840 (65.2)	15 (1.2)	433 (33.6)
	a. Yes (1 point)	b. No (0 points)	c. Uncertain (0 points)
Are you familiar with beverages that have high sugar content?	885 (68.7)	131 (10.2)	272 (21.1)
	a. Yes (0 points)	b. No (1 point)	c. Uncertain (0 points)
Is consuming sugar-free beverages, which substitute white sugar, brown sugar, cane sugar, glucose, etc., with “artificial sweeteners” on the market, therefore, have no impact on human health?	147 (11.4)	883 (68.6)	258 (20.0)
	a. Yes (1 point)	b. No (0 points)	c. Uncertain (0 points)
Do calorie-free, sugar-free carbonated beverages increase the risk of hyperuricemia?	461 (35.8)	164 (12.7)	663 (51.5)

**Table 3:** Responses to items in the participant knowledge dimension (Multiple choices allowed)

Item	Option	n (%)
Which of the following are common sugary beverages? (Multiple choices allowed)	a. Cola/Sprite	1246 (96.7)
	b. Fruit juice drinks	1190 (92.4)
	c. Bubble tea	1223 (95.0)
	d. Americano coffee	283 (22.0)
	e. Sports drinks (e.g., Red Bull, Powerade)	896 (69.6)
	f. Tea-based drinks (e.g., Kangshifu green tea, iced black tea)	902 (70.0)
	g. Uncertain	11 (0.9)
What are the potential harms of sugary beverages to the human body? (Multiple choices allowed)	a. Obesity	1263 (98.1)
	b. Tooth decay	1175 (91.2)
	c. Cardiovascular diseases	1108 (86.0)
	d. Hyperuricemia	995 (77.3)
	e. Accelerated aging	1024 (79.5)
	f. Uncertain	12 (0.9)

The attitude score was 22 (20, 24) out of 30 points (73.3%). The distribution of responses for each item in the attitude section is presented in Tables 4 and 5. The mean practice score was 27.5 (23, 31.75) out of 40 points (68.75%). The distribution of responses for each item in the practice section is provided in Table 6.

**Table 4:** Responses to the items in the participant attitude dimension

	a. Significantly impactful (5 points)	b. Moderately impactful	c. Uncertain	d. Not very impactful	e. No impact at all (1 point)
What do you believe is the impact of frequent consumption of sugary beverages on health?	671 (52.0)	515 (40.0)	69 (5.4)	29 (2.3)	4 (0.3)
	a. Numerous factors contribute to hyperuricemia, and sugary beverages are insignificant (1 point)	b. Numerous factors contribute to hyperuricemia, and sugary beverages play a role	c. Uncertain, varies by individual	d. Excessive sugary beverage intake might be a factor leading to hyperuricemia	e. Excessive sugary beverage intake is a significant factor causing hyperuricemia (5 points)
Do you think the occurrence of hyperuricemia is related to the consumption of sugary beverages?	66 (5.1)	529 (41.1)	318 (24.7)	213 (16.5)	162 (12.6)
	a. Strongly agree 1 point)	b. Somewhat agree	c. Uncertain	d. Somewhat disagree	e. Strongly disagree (5 points)
Do you think you are in good health and unlikely to develop hyperuricemia easily?	100 (7.7)	238 (18.5)	407 (31.6)	363 (28.2)	180 (14.0)
	a. Very concerned, harmful to health (5 points)	b. Concerned, affects health	c. Neutral	d. Not very concerned, treatable	e. Completely unconcerned, a minor issue (1 point)
If you had hyperuricemia, would you be worried?	446 (34.6)	650 (50.5)	140 (10.9)	39 (3.0)	13 (1.0)
	a. Strongly agree, sugary beverages are entirely harmful (5 points)	b. Agree, sugary beverages impact health	c. Neutral	d. Somewhat disagree, drinking a little is okay	e. Completely disagree, excessive consumption doesn’t have a big impact (1 point)
Do you think you should reduce the consumption of sugary beverages?	465 (36.1)	667 (51.8)	110 (8.5)	44 (3.4)	2 (0.2)
	a. Very difficult to control, actively want to drink beverages (1 point)	b. Somewhat difficult to control, unintentionally consume (at social events, when thirsty)	c. Uncertain, depends on the situation	d. Can generally control	e. Can strictly control, constantly remind myself (5 points)
Do you find it challenging to control reducing or not drinking sugary beverages?	150 (11.7)	357 (27.7)	113 (8.8)	499 (38.7)	169 (13.1)

**Table 5:** Responses to the items in the participant attitude dimension (Multiple choices allowed)

	a. Influenced by people around me	b. Hot weather, drinking cold beverages for cooling	c. As afternoon tea, to replenish energy	d. Sold everywhere, convenient to purchase	e. Most beverages are sugary, can’t find non-sugar options	f. Good taste, mood regulation	g. Other
What factors do you believe would make you choose sugary beverages?	102(7.9)	526(40.8)	55(4.3)	59(4.6)	110(8.5)	398(30.9)	38(3.0)

**Table 6:** Responses to items in the participant practice dimension

		a. Yes (2.5 points)		b. No			c. Uncertain					
Have you paid attention to hyperuricemia?		628 (48.8)		594 (46.1)			66 (5.1)					
		a. Yes (2.5 points)		b. No			c. Uncertain					
Have you paid attention to the relationship between sugar and hyperuricemia?		419 (32.5)		774 (60.1)			95 (7.4)					
		a. Multiple times a day (1 point)		b. Once a day			c. Several times a week		d. Several times a month		e. Rarely or seldom drink sugary beverages (5 points)	
How often do you consume sugary beverages?		59 (4.6)		137 (10.6)			433 (33.6)		379 (29.4)		280 (21.8)	
		a. Very inclined (5 points)		b. Moderately inclined			c. Uncertain		d. Not very inclined		e. Not inclined, only care about taste (1 point)	
Are you more inclined towards sugar-free or low-sugar beverages when choosing drinks?		464 (36.0)		415 (32.2)			109 (8.5)		138 (10.7)		162 (12.6)	
		a. Frequently attentive (5 points)		b. Occasionally attentive			c. Uncertain		d. Not very attentive		e. Not attentive (1 point)	
When purchasing beverages, do you pay attention to the ingredient list?		391 (30.4)		500 (38.8)			38 (3.0)		249 (19.3)		110 (8.5)	
		a. Tried and maintained for a long time (5 points)		b. Tried but only maintained for a short period			c. Tried but couldn’t control and ended up drinking		d. Haven’t tried		e. Not planning to or don’t want to reduce sugar beverage intake (1 point)	
Have you attempted to reduce the consumption of sugary beverages?		529 (41.1)		370 (28.7)			161 (12.5)		197 (15.3)		31 (2.4)	
		a. Often advised by others		b. Occasionally advised by others			c. Rarely advised by others		d. Never advised by others		e. I don’t drink sugary beverages	
Has anyone advised you to drink less sugary beverages?		391 (30.4)		495 (38.4)			209 (16.2)		100 (7.8)		93 (7.2)	
		a. I wouldn’t, relatively expensive and inconvenient (1 point)		b. Not really, a bit of a hassle			c. Uncertain, depends on the situation		d. Generally will, fresh-squeezed juice is fresher		e. Definitely will, health is most important (5 points)	
Would you choose freshly squeezed juice over prepared fruit juice?		210 (16.3)		319 (24.8)			343 (26.6)		291 (22.6)		125 (9.7)	
		a. Not concerned, irrelevant to me (1 point)		b. Not very concerned, never heard of it			c. Uncertain		d. Occasionally concerned, might inadvertently see relevant reports		e. Frequently concerned, because I prioritize health (5 points)	
Do you pay attention to news about the harm of sugary beverages to the human body?		52 (4.0)		178 (13.8)			116 (9.0)		731 (56.8)		211 (16.4)	
		a. No (5 points)		b. Rarely			c. Uncertain		d. Maybe, some beverages are relatively healthy		e. Yes, convenient to drink (1 point)	
Does hyperuricemia requiring more urination lead you to drink more beverages for increased urination?		650 (50.5)		295 (22.9)			207 (16.1)		100 (7.8)		36 (2.7)	
	a. Soda		b. Milk		c. Fruit juice drinks	d. Energy drinks		e. Tea-based drinks		f. Water		g. Other
What beverage do you tend to choose when thirsty?	176 (13.7)		42 (3.3)		92 (7.1)	48 (3.7)		211 (16.4)		666 (51.7)		53 (4.1)

Significant differences were found in attitude and practice scores across different age groups (*P* < 0.001). There were statistically significant differences in knowledge and practice scores across varying levels of education (*P* < 0.05), with higher education correlating with higher knowledge scores. Different occupational categories yielded statistically significant variations in knowledge, attitude, and practice scores (*P* < 0.001), with healthcare workers having the highest scores across dimensions. Single children demonstrated higher knowledge and practice levels than children with siblings (*P* < 0.05). Distinct weight categories also exhibited statistically significant differences in KAP scores (*P* < 0.05), with the overweight group displaying higher knowledge and attitude scores. Participants with hyperuricemia scored significantly higher in knowledge, attitudes, and practices than those without gout (*P* < 0.001). Participants with gout also achieved significantly higher scores in all dimensions than those without gout (*P* < 0.001).

### Correlations

The correlation analysis reveals that the knowledge scores were positively correlated with the attitude (*r* = 0.326, *P* < 0.001) and practice (*r* = 0.485, *P* = 0.001) scores, while the attitude scores were positively correlated with the practice scores (*r* = 0.448, *P* < 0.001) (Table 7).

**Table 7:** Correlation of scores among KAP dimensions

	Knowledge	Attitude	Practice
Knowledge	1.000	/	/
Attitude	0.326 (*P* < 0.001)	1.000	/
Practice	0.485 (*P* < 0.001)	0.448 (*P* < 0.001)	1.000

#### Univariable and multivariable analyses

The multivariable analysis showed that bachelor’s/associate education (OR = 1.912, 95%CI: 1.128–3.239, *P* = 0.016), white collar/employee (OR = 0.147, 95%CI: 0.105–0.206, *P* < 0.001), educator (OR = 0.300, 95%CI: 0.174–0.518, *P* < 0.001), healthcare worker (OR = 0.277, 95%CI: 0.188–0.407, *P* < 0.001), not suffering from hyperuricemia (OR = 0.386, 95%CI: 0.253–0.590, *P* < 0.001), and not having gout (OR = 0.456, 95%CI: 0.282–0.736, *P* = 0.001) were independently associated with knowledge (Table 8).

**Table 8:** Logistic regression analysis of the knowledge dimension

Cut-off value: ≥16/<16	n	Univariable		Multivariable (regression method: enter)	
		OR (95%CI)	*P*	OR (95%CI)	*P*
Gender					
Male	294/559	ref.			
Female	405/729	1.127 (0.903, 1.406)	0.290		
Age					
≤ 25years	143/283	ref.			
26–30 years	173/296	1.377 (0.992, 1.912)	0.056		
31–35 years	142/249	1.107 (0.820, 1.494)	0.506		
36–40 years	160/296	0.949 (0.727, 1.239)	0.699		
41–44 years	81/164	0.797 (0.574, 1.107)	0.176		
Education level					
High school or below	27/84	ref.		ref.	
Bachelor/Associate	402/731	2.580 (1.595, 4.171)	< 0.001	1.912 (1.128, 3.239)	0.016
Postgraduate or above	270/473	1.748 (1.293, 2.363)	< 0.001	1.286 (0.916, 1.805)	0.146
Occupation					
Student	274/349	ref.		ref.	
White-collar/employee	197/476	0.193 (0.141, 0.265)	< 0.001	0.147 (0.105, 0.206)	< 0.001
Educator	43/75	0.368 (0.218, 0.621)	< 0.001	0.300 (0.174, 0.518)	< 0.001
Healthcare worker	100/203	0.266 (0.183, 0.387)	< 0.001	0.277 (0.188, 0.407)	< 0.001
Other	85/185	0.233 (0.158, 0.342)	< 0.001	0.175 (0.115, 0.267)	< 0.001
Are you the only child?					
Yes	345/600	ref.		ref.	
No	354/688	0.783 (0.628, 0.977)	0.030	0.824 (0.644, 1.054)	0.123
Weight status					
Underweight	69/145	ref.			
Normal weight	320/601	1.254 (0.872, 1.804)	0.221		
Overweight	209/380	1.202 (0.916, 1.577)	0.184		
Obese	101/162	1.531 (1.083, 2.166)	0.016		
Do you suffer from hyperuricemia?					
Yes	189/253	ref.		ref.	
No	510/1035	0.329 (0.242, 0.448)	< 0.001	0.386 (0.253, 0.590)	< 0.001
Do you suffer from gout?					
Yes	134/183	ref.		ref.	
No	565/1105	0.383 (0.270, 0.542)	< 0.001	0.456 (0.282, 0.736)	0.001

Age 26–30 (OR = 1.470, 95%CI: 1.052–2.052, *P* = 0.024), age 31–35 (OR = 1.489, 95%CI: 1.097–2.022, *P* = 0.011), age 36–40 (OR = 0.328, 95%CI: 1.010–1.746, *P* = 0.042), age 41–44 (OR = 1.548, 95%CI: 1.091–2.198, *P* = 0.014), and not having hyperuricemia (OR = 0.512, 95%CI: 0.345–0.760, *P* = 0.001) were independently associated with attitude (Table 9).

**Table 9:** Logistic regression analysis of the attitude dimension

Cut-off value: ≥22/<22	n	Univariable		Multivariable (regression method: enter)	
		OR (95%CI)	*P*	OR (95%CI)	*P*
Gender					
Male	302/559	ref.			
Female	404/729	1.058 (0.848, 1.320)	0.619		
Age					
≤ 25years	118/283	ref.		ref.	
26–30 years	159/296	1.623 (1.168, 2.254)	0.004	1.470 (1.052, 2.052)	0.024
31–35 years	146/249	1.556 (1.151, 2.102)	0.004	1.489 (1.097, 2.022)	0.011
36–40 years	175/296	1.370 (1.046, 1.794)	0.022	1.328 (1.010, 1.746)	0.042
41–44 years	108/164	1.689 (1.197, 2.382)	0.003	1.548 (1.091, 2.198)	0.014
Education level )					
High school or below	50/84	ref.			
Bachelor/associate	395/731	0.799 (0.505, 1.265)	0.339		
Postgraduate or above	261/473	0.936 (0.699, 1.255)	0.659		
Occupation					
Student	207/349	ref.			
White-collar/employee	255/476	0.792 (0.599, 1.047)	0.101		
Educator	40/75	0.784 (0.475, 1.294)	0.341		
Healthcare worker	85/203	0.494 (0.348, 0.702)	< 0.001		
Other	119/185	1.237 (0.855, 1.789)	0.259		
Are you the only child?					
Yes	328/600	ref.			
No	378/688	1.011 (0.812, 1.260)	0.921		
Weight status					
Underweight	71/145	ref.			
Normal weight	340/601	1.358 (0.944, 1.953)	0.099		
Overweight	204/380	1.037 (0.790, 1.360)	0.794		
Obese	91/162	1.133 (0.806, 1.591)	0.472		
Do you suffer from hyperuricemia?					
Yes	184/253	ref.		ref.	
No	522/1035	0.382 (0.282, 0.516)	< 0.001	0.512 (0.345, 0.760)	0.001
Do you suffer from gout?					
Yes	135/183	ref.		ref.	
No	571/1105	0.380 (0.268, 0.539)	< 0.001	0.689 (0.436, 1.089)	0.111

White collar/employee (OR = 0.386, 95%CI: 0.285–0.521, *P* < 0.001), educator (OR = 0.534, 95%CI: 0.317–0.899, *P* = 0.018), healthcare worker (OR = 0.341, 95%CI: 0.236–0.493, *P* < 0.001), having siblings (OR = 0.725, 95%CI: 0.573–0.917, *P* = 0.007), and not suffering from hyperuricemia (OR = 0.442, 95%CI: 0.296–0.659, *P* < 0.001), were independently associated with practice (Table 10).

**Table 10:** Logistic regression analysis of the Practice dimension

Cut-off value: ≥27.5/<27.5	n	Univariable		Multivariable (regression method: enter)	
		OR (95%CI)	*P*	OR (95%CI)	*P*
Gender					
Male	273/559	ref.			
Female	373/729	1.098 (0.880, 1.368)	0.407		
Age					
≤ 25years	110/283	ref.			
26–30 years	151/296	1.638 (1.177, 2.279)	0.003		
31–35 years	135/249	1.455 (1.079, 1.963)	0.014		
36–40 years	147/296	1.070 (0.820, 1.396)	0.619		
41–44 years	103/164	1.800 (1.284, 2.524)	0.001		
Education level					
High school or below	30/84	ref.		ref.	
Bachelor/associate	371/731	1.855 (1.160, 2.966)	0.010	1.470 (0.895, 2.414)	0.128
Postgraduate or above	245/473	1.420 (1.056, 1.909)	0.020	1.173 (0.851, 1.617)	0.331
Occupation					
Student	225/349	ref.		ref.	
White-collar/employee	215/476	0.454 (0.342, 0.603)	< 0.001	0.386 (0.285, 0.521)	< 0.001
Educator	40/75	0.630 (0.381, 1.042)	0.072	0.534 (0.317, 0.899)	0.018
Healthcare worker	77/203	0.337 (0.235, 0.482)	< 0.001	0.341 (0.236, 0.493)	< 0.001
Other	89/185	0.511 (0.356, 0.734)	< 0.001	0.427 (0.289, 0.631)	< 0.001
Are you the only child?					
Yes	331/600	ref.		ref.	
No	315/688	0.686 (0.551, 0.855)	0.001	0.725 (0.573, 0.917)	0.007
Weight status					
Underweight	53/145	ref.			
Normal weight	321/601	1.990 (1.369, 2.893)	< 0.001		
Overweight	190/380	1.231 (0.935, 1.619)	0.139		
Obese	82/162	1.177 (0.839, 1.652)	0.346		
Do you suffer from hyperuricemia?					
Yes	174/253	ref.		ref.	
No	472/1035	0.381 (0.284, 0.510)	< 0.001	0.442 (0.296, 0.659)	< 0.001
Do you suffer from gout?					
Yes	121/183	ref.		ref.	
No	525/1105	0.464 (0.334, 0.644)	< 0.001	0.719 (0.455, 1.135)	0.156

## Discussion

The prevalence of hyperuricemia in China has been consistently increasing, particularly among the younger generation [6]. The excessive consumption of sugar-sweetened beverages is associated with hyperuricemia [12, 13]. This study examined the KAP of Chinese young adults regarding sugar-sweetened beverage consumption and the correlation with hyperuricemia. The results suggest that Chinese young adults display moderate KAP toward sugar-sweetened beverages. Notably, the participants with hyperuricemia exhibited significantly higher KAP scores than their counterparts without hyperuricemia. The results suggest the influence of passive learning or patient education among young adults. Furthermore, a proper KAP toward sugar-sweetened is necessary to prevent hyperuricemia and subsequent complications. It calls for subsequent efforts to enhance proactive engagement and education on relevant content.

The present study revealed moderate KAP toward sugar-sweetened beverages among Chinese young adults (18–44 years). The participants were mostly female, highly educated, workers, and with a normal body weight. In contrast, a study from Malaysia showed good knowledge, moderate attitude, and poor practice toward sugar-sweetened beverages among adolescents; higher body fat was associated with a more favorable attitude, while being female, having a low income, being 16–17 years old, and being from urban areas were associated with better KAP [24]. Encouragingly, in the present study, half of the participants were understanding the predisposing factors of hyperuricemia, However, it is worth noting that a small proportion of participants did not know whether sugar-sweetened beverage intake is associated with hyperuricemia. This knowledge gap may be attributed, in part, to the inadequate or unclear delivery of health-related information. The observed weak correlation between knowledge and attitudes could be attributed to the undeniable allure of sugar-sweetened beverages, which, due to their delightful taste, may overshadow concerns regarding their adverse impact on health. In addition, sugar-sweetened beverages potentially have addictive effects [25], and such addiction could preclude individuals from seeing harm in such beverages. Still, having a proper practice toward sugar-sweetened beverages is important as the consumption of these beverages over the long-term will have detrimental effects on health, including hyperuricemia, dyslipidemia, and hyperglycemia and their complications such as chronic kidney diseases, type 2 diabetes mellitus, and cardiovascular diseases [10, 11]. The female gender was not associated with the KAP scores. Indeed, females are generally more concerned about body appearance [26–28], but older age was independently associated with higher attitude scores, as supported by an Australian study [26]. In addition, before menopause, women are protected by estrogens against the development of hyperuricemia and other metabolic disorders [29–31], possibly making younger women adopt a more casual attitude toward hyperuricemia. As individuals age, they can become more concerned with their health.

In the present study, a higher socioeconomic status was associated with higher KAP scores. A higher socioeconomic status has been associated with higher health literacy [32]. Socioeconomic status is also a major contributor to the knowledge about nutrition [33, 34]. It should be emphasized that the majority of the participants were highly educated (56.8% with a bachelor’s degree; 36.6% with postgraduate and above studies). Moreover, around 32.9% of the participants were healthcare workers (27.1%) and educators (5.8%). Although the present study showed that a higher socioeconomic status was associated with higher KAP scores, the study population was biased toward a population with more favorable health literacy, which probably overestimated the results. Nevertheless, a major factor to consider is that unhealthy foods are inexpensive, while healthy foods are expensive; therefore, a lower socioeconomic status can be associated with poorer practice, but not always as per the individual’s choice [35]. Furthermore, all three KAP dimensions were positively correlated to each other, meaning that improving knowledge should also improve attitude and practice. Indeed, according to the KAP theory, knowledge is the basis for practice, while attitude is the force driving practice [14, 15]. Hence, stakeholders should design and implement educational interventions to improve the knowledge of Chinese young adults about sugar-sweetened beverages. Such educational programs should include the types of sugar-sweetened beverages, the risks associated with sugar-sweetened beverages, and how to adopt appropriate lifestyle habits to avoid sugar-sweetened beverages. The programs should target everyone in the society. To do so, the programs should be available on every platform available, including the Internet, TV, radio, schools, hospitals, and workplaces. It could take the form of brief information about general knowledge and good lifestyle habits, with an invitation to consult healthcare providers.

The present study also has limitations. The frequencies of gout were high because many participants were enrolled at a gout clinic. The information collected via the questionnaire in the present study is online and self-reported; thus, the results might be subject to inaccuracy. The cross-sectional study prevented any causality inference. In addition, the results were from a single point in time, but they could serve as a historical control to examine the impact of future interventions. The questionnaire was designed by local investigators and had to reflect the local reality of the participants. Furthermore, the questionnaire could be influenced by local practices, guidelines, and policies. All KAP studies are at risk of social desirability bias. It entails that a participant could be tempted to answer what they know they should do instead of what they are doing [36, 37]. Considering that the attitude and practice levels were about the same as the knowledge level, that bias is possible.

## Conclusion

In conclusion, Chinese young adults display moderate KAP toward sugar-sweetened beverages. Notably, an association was observed between hyperuricemia and each KAP dimension. The consumption of sugar-sweetened beverages is part of bad lifestyle habits. The results should guide the design of educational programs to improve the knowledge of the health risks associated with sugar-sweetened beverages and encourage individuals to control their consumption of sugar-sweetened beverages.

### Electronic supplementary material

Below is the link to the electronic supplementary material.

Supplementary Material 1

## Data Availability

All data generated or analyzed during this study are included in this article.

## Abbreviations

KAP: knowledge, attitudes and practices
