# Supplementary material for: Assessment of the correlation between KAP scores regarding sugar-sweetened beverage consumption and hyperuricemia amongst Chinese young adults
Source: BMC Public Health. 2024 Apr 17;24:1074. doi: 10.1186/s12889-024-18513-x (PMC11025163; doi:10.1186/s12889-024-18513-x)
Supplement: Supplementary file 1 — Supplementary Material 1 [file 12889_2024_18513_MOESM1_ESM.doc]

Questionnaire ID:

| Dear Friend,  We are healthcare professionals from Peking Union Medical College Hospital's Gout Clinic, cordially inviting you to participate in our research project. This study aims to comprehend the correlation between Knowledge, Attitude, and Practice (KAP) scores regarding sugary beverage consumption and hyperuricemia among young individuals in China. This endeavour seeks to provide a foundation for developing evidence-based early intervention strategies, potentially contributing to the enhancement of patients' health in the future. Your participation in this study is voluntary. Should you decide to partake, please review the following instructions.  1. Kindly complete the questionnaire. Your responses are not graded as right or wrong; rather, we request that you provide accurate information based on your actual experiences. Should any queries arise during the process, feel free to reach out to us. Upon completion, kindly submit the questionnaire in a timely manner.  2. This study involves a straightforward questionnaire survey. It will not cause any harm to your physical or psychological well-being. However, it may touch upon personal matters such as gender and age. Please be assured that your information will be treated with the utmost confidentiality and will not be disclosed.  3. As a participant, you can stay informed about the study's progress and related information. Should you decide to withdraw from the study, kindly inform us. Your data will not be included in the research outcomes.  Finally, we sincerely appreciate your willingness to dedicate your valuable time to support our scientific research!  □ I have understood and agreed that the collected data will be used for scientific research purposes.  Informed Consent Signature：  Participation Date：      Year Month Day |
| --- |
|  |

**Assessment of the Correlation Between Sugary Beverage Consumption KAP Scores and Hyperuricemia Among Young Adults in China**

**Part 1: Sociodemographic Information**

1. Your gender：
   1. Male
   2. Female
2. Your age：

a. Under 15 years

b.15~18 years

c.19~25 years

d.26~30 years

e.31~35 years

f.36~40 years

e.41-44 years

f. Above 44 years

1. Your education level：

a. Primary school

b. Junior high school

c. High school

d. Bachelor's degree/Associate degree

e. Postgraduate degree or above

1. Your occupation：
   1. Student
   2. White-collar/Company employee
   3. Educator
   4. Healthcare professional
   5. Other (please specify)
2. Are you the only child in your family?
   1. Yes
   2. No
3. Weight status：
   1. Underweight
   2. Normal weight
   3. Overweight
   4. Obese
4. Do you have any of the following conditions? (Multiple choices allowed)
   1. Hyperglycemia
   2. Hypertension
   3. Hyperlipidemia
   4. None
5. Do you suffer from hyperuricemia?
   1. Yes
   2. No
6. Do you suffer from gout?
7. Yes
8. No

**Part 2: Survey on Sugary Beverage Consumption and Hyperuricemia Knowledge Among Chinese Young Adults**

1. Have you heard of hyperuricemia?
   1. Yes
   2. No
   3. Uncertain
2. Are you aware of the triggering factors of hyperuricemia?
3. Yes
4. No
5. Uncertain
6. Do you comprehend the risks associated with hyperuricemia?
7. Yes
8. No
9. Uncertain
10. Is the intake of sugary beverages related to hyperuricemia?
    1. Yes
    2. No
    3. Uncertain
11. Is fructose in sugary beverages a significant factor in causing hyperuricemia?
    1. Yes
    2. No
    3. Uncertain
12. Are low purine levels in sugary beverages unlikely to cause hyperuricemia?
    1. Yes
    2. No
    3. Uncertain
13. Is hyperuricemia closely related to the onset of gout?
    1. Yes
    2. No
    3. Uncertain
14. Does hyperuricemia increase the risk of developing diabetes?
    1. Yes
    2. No
    3. Uncertain
15. Can severe hyperuricemia lead to acute kidney failure?
    1. Yes
    2. No
    3. Uncertain
16. Are you familiar with beverages that have high sugar content?
    1. 清楚
    2. Uncertain
    3. Uncertain
17. Which of the following are common sugary beverages? (Multiple choices allowed)
    1. Cola/Sprite
    2. Fruit juice drinks
    3. Bubble tea
    4. Americano coffee
    5. Sports drinks (e.g., Red Bull, Powerade)
    6. Tea-based drinks (e.g., Kangshifu green tea, iced black tea)
    7. Uncertain
18. What are the potential harms of sugary beverages to the human body? (Multiple choices allowed)
    1. Obesity
    2. Tooth decay
    3. Cardiovascular diseases
    4. Hyperuricemia
    5. Accelerated aging
    6. Uncertain
19. Is the consumption of sugar-free beverages, which substitute white sugar, brown sugar, cane sugar, glucose, etc., with "artificial sweeteners" on the market, therefore, have no impact on human health?
    1. Yes
    2. No
    3. Uncertain
20. Does calorie-free, sugar-free carbonated beverages increase the risk of hyperuricemia?
    1. Yes
    2. No
    3. Uncertain

**Part 3: Survey on Attitudes Towards Sugary Beverage Consumption and Hyperuricemia Among Chinese Young Adults**

1. What do you believe is the impact of frequent consumption of sugary beverages on health?
   1. Significantly Impactful
   2. Moderately Impactful
   3. Uncertain
   4. Not Very Impactful
   5. No Impact at All
2. Do you think the occurrence of hyperuricemia is related to the consumption of sugary beverages?
3. Numerous factors contribute to hyperuricemia, and sugary beverages are insignificant
4. Numerous factors contribute to hyperuricemia, and sugary beverages play a role
5. Uncertain, varies by individual
6. Excessive sugary beverage intake might be a factor leading to hyperuricemia
7. Excessive sugary beverage intake is a significant factor causing hyperuricemia
8. Do you think that you are in good health and unlikely to develop hyperuricemia easily?
   1. Strongly agree
   2. Somewhat agree
   3. Uncertain
   4. Somewhat disagree
   5. Strongly disagree
9. If you had hyperuricemia, would you be worried?
   1. Very concerned, harmful to health
   2. Concerned, affects health
   3. Neutral
   4. Not very concerned, treatable
   5. Completely unconcerned, a minor issue
10. Do you think you should reduce the consumption of sugary beverages?
    1. Strongly agree, sugary beverages are entirely harmful
    2. Agree, sugary beverages impact health
    3. Neutral
    4. Somewhat disagree, drinking a little is okay
    5. Completely disagree, excessive consumption doesn't have a big impact
11. Do you find it challenging to control reducing or not drinking sugary beverages?
    1. Very difficult to control, actively want to drink beverages
    2. Somewhat difficult to control, unintentionally consume (at social events, when thirsty)
    3. Uncertain, depends on the situation
    4. Can generally control
    5. Can strictly control, constantly remind myself
12. What factors do you believe would make you choose sugary beverages?
    1. Influenced by people around me
    2. Hot weather, drinking cold beverages for cooling
    3. As afternoon tea, to replenish energy
    4. Sold everywhere, convenient to purchase
    5. Most beverages are sugary, can't find non-sugar options
    6. Good taste, mood regulation
    7. Other factors

**Part 4: Survey on Practices Regarding Sugary Beverage Consumption and Hyperuricemia Among Chinese Young Adults**

1. Have you paid attention to hyperuricemia?
   1. Yes
   2. No
   3. Uncertain
2. Have you paid attention to the relationship between sugar and hyperuricemia?
   1. Yes
   2. No
   3. Uncertain
3. How often do you consume sugary beverages?
   1. Multiple times a day
   2. Once a day
   3. Several times a week
   4. Several times a month
   5. Rarely or almost never drink sugary beverages
4. What beverage do you tend to choose when thirsty?
   1. Soda
   2. Milk
   3. Fruit juice drinks
   4. Energy drinks
   5. Tea-based drinks
   6. Water
   7. Other
5. Are you more inclined towards sugar-free or low-sugar beverages when choosing drinks?
   1. Very inclined
   2. Moderately inclined
   3. Uncertain
   4. Not very inclined
   5. Not inclined, only care about taste
6. When purchasing beverages, do you pay attention to the ingredient list?
   1. Frequently attentive
   2. Occasionally attentive
   3. Uncertain
   4. Not very attentive
   5. Not attentive
7. Have you attempted to reduce the consumption of sugary beverages?

a. Tried and maintained for a long time

b. Tried but only maintained for a short period

c. Tried but couldn't control and ended up drinking

d. Haven't tried

e. Not planning to or don't want to reduce sugar beverage intake

1. Has anyone advised you to drink less sugary beverages?
   1. Often advised by others
   2. Occasionally advised by others
   3. Rarely advised by others
   4. Never advised by others
   5. I don't drink sugary beverages
2. Would you choose freshly squeezed juice over prepared fruit juice?
   1. I wouldn’t, relatively expensive and inconvenient
   2. Not really, a bit of a hassle
   3. Uncertain, depends on the situation
   4. Generally will, fresh-squeezed juice is fresher
   5. Definitely will, health is most important
3. Do you pay attention to news about the harm of sugary beverages to the human body?
   1. Not concerned, irrelevant to me
   2. Not very concerned, never heard of it
   3. Uncertain
   4. Occasionally concerned, might inadvertently see relevant reports
   5. Frequently concerned, because I prioritize health
4. Does hyperuricemia requiring more urination lead you to drink more beverages for increased urination?
   1. No
   2. Rarely
   3. Uncertain
   4. Maybe, some beverages are relatively healthy
   5. Yes, convenient to drink
